# The Impact of Personality Factors and Preceding User Comments on the Processing of Research Findings on Deep Brain Stimulation: A Randomized Controlled Experiment in a Simulated Online Forum

**DOI:** 10.2196/jmir.4382

**Published:** 2016-03-03

**Authors:** Insa Feinkohl, Danny Flemming, Ulrike Cress, Joachim Kimmerle

**Affiliations:** ^1^Leibniz-Institut für Wissensmedien | Knowledge Media Research CenterKnowledge Construction LabTuebingenGermany; ^2^Department of PsychologyUniversity of TuebingenTuebingenGermany

**Keywords:** medical news, online forum, scientific literacy, epistemological beliefs, academic self-efficacy, tentativeness

## Abstract

**Background:**

Laypeople frequently discuss medical research findings on Web-based platforms, but little is known about whether they grasp the tentativeness that is inherent in these findings. Potential influential factors involved in understanding medical tentativeness have hardly been assessed to date.

**Objective:**

The research presented here aimed to examine the effects of personality factors and of other users’ previous contributions in a Web-based forum on laypeople’s understanding of the tentativeness of medical research findings, using the example of research on deep brain stimulation.

**Methods:**

We presented 70 university students with an online news article that reported findings on applying deep brain stimulation as a novel therapeutic method for depression, which participants were unfamiliar with. In a randomized controlled experiment, we manipulated the forum such that the article was either accompanied by user comments that addressed the issue of tentativeness, by comments that did not address this issue, or the article was accompanied by no comments at all. Participants were instructed to write their own individual user comments. Their scientific literacy, epistemological beliefs, and academic self-efficacy were measured. The outcomes measured were perceived tentativeness and tentativeness addressed in the participants’ own comments.

**Results:**

More sophisticated epistemological beliefs enhanced the perception of tentativeness (standardized β=.26, *P*=.034). Greater scientific literacy (stand. β=.25, *P*=.025) and greater academic self-efficacy (stand. β=.31, *P*=.007) were both predictors of a more extensive discussion of tentativeness in participants’ comments. When forum posts presented in the experiment addressed the issue of tentativeness, participants’ subsequent behavior tended to be consistent with what they had read in the forum, *F*_2,63_=3.66; *P=*.049, η_p_^2^=.092.

**Conclusions:**

Students’ understanding of the tentativeness of research findings on deep brain stimulation in an online forum is influenced by a number of character traits and by the previous comments that were contributed to the forum by other users. There is potential for targeted modification of traits such as scientific literacy, epistemological beliefs, and academic self-efficacy to foster critical thinking in laypeople who take part in online discussions of medical research findings.

## Introduction

The resources made available to medical research have seen a steady increase over the past century [1,2]. Health and disease are better understood than ever, and novel technologies for diagnosis and therapy are continuously being developed. The mass media landscape has undergone a kind of evolution that parallels this development. Following what has been termed “scientific malnutrition” during the 20th century [3], mainstream media have increasingly begun to report on findings from medical research studies [4,5]. With the rise of online media and subsequently of Web 2.0, which allows readers to “share” news items among their associates, the global distribution of health news items now reaches vast dimensions [6,7].

Like all scientific research, that in the medical domain is affected by the inherently uncertain, temporary, and revisionary nature of scientific findings [8,9], which is referred to in the literature as the “tentativeness” [8,10,11] of science. This tentativeness is characterized by the fact that research findings are frequently quite controversial and that they are contradictory or inconsistent [8,10]. Usually, scientific findings are provisional and they cannot readily be generalized.

When confronted with medical research findings in the media, it is important for non-professionals to detect and to understand this tentativeness [10,12] because the perception of medical research has an impact on making personal decisions related to health. Coverage of health-related topics in the media, for instance, is correlated with the frequency of online searches for that topic [13]. Understanding and appreciating the tentativeness of medical research findings are important for applying critical thinking to the medical context [14].

### The Impact of Web 2.0 on Perceiving Media Content

Laypeople’s understanding of tentativeness has gained relevance with the rise of Web 2.0. With the term laypeople, we refer to all Internet users who are not medical experts but nevertheless are interested in reading and understanding online newspaper texts about medical topics. So with laypeople, we mean casual readers of science journalistic texts who do not necessarily have to be patients or individuals with the same medical condition as described in the text. Readers of science journalistic articles often have an academic background and are therefore not representative of the average population. The role of laypeople has changed, since they are no longer mere recipients of information from journalists. Nowadays, anybody with Internet access may receive medical information and personal opinions from other non-professionals [15-17]. Facilitated by the commenting functions on Internet platforms such as online news outlets and social media websites, laypeople have the opportunity to become active producers of medical media content [18,19].

Since evidence suggests that about 85% of those people who read online news articles also read associated user comments [20], readers’ decisions related to health are likely to be influenced not only by the article itself, but additionally by user comments. In contrast to news items, these sources are not subject to any formal gatekeeping [21], and so users have to independently evaluate the unfiltered information in order to decide whether to accept or reject its content [8]. Consequently, research into the processes that are at play in the interaction of laypeople on online platforms is essential, particularly in regard to the extent to which the tentativeness of medical research findings is grasped. But currently such research lags way behind the continuous technological advancement of Web 2.0. Interindividual determinants of critical thinking in the context of medical information in particular are under-researched, despite the fact that such determinants have been identified previously in conventional environments [22].

### Determinants of Understanding Tentativeness

Factors related to scientific understanding and scientific knowledge are known to have an impact on people’s ability to critically deal with scientific information. Accordingly, we assume that such factors may also play a role when it comes to understanding the tentativeness of medical research findings presented in the media. One such factor is scientific literacy [23], previously defined as the ability to “use evidence and data to evaluate the quality of science information and arguments put forth by scientists and in the media” [24], or as having “a basic vocabulary of scientific terms and constructs; and […] a general understanding of the nature of scientific inquiry” [25]. Accordingly, a range of tests is available to tap a person’s scientific literacy. One approach is to assess their ability to understand the Tuesday science section of the *New York Times* [25]; another is to use open-ended questions such as “What is a molecule?” Using the latter approach, Miller [26] found that scientific literacy among adults has seen a substantial increase during the latter part of the 20th century. It is even higher among those in the younger generation, which suggests a future trend for continuation of the improvement in scientific literacy in the population [25]. At the same time, however, evidence suggests that intervention programs targeting this ability may be of only limited success [27]. On the basis that scientific literacy is closely related to critical thinking aptitude [28], its effect on the ability to understand tentativeness in medical research is plausible from a theoretical and empirical point of view.

Another factor that has an impact on how critically people handle scientific information and that, as a consequence, is supposed to influence whether they grasp the tentativeness of medical research findings is a person’s beliefs about the nature of knowledge, which is referred to in the literature as epistemological beliefs [29-31]. People align on a spectrum spanning simple epistemological beliefs, meaning beliefs that knowledge is static and absolute, to sophisticated epistemological beliefs that see knowledge as a complex and dynamic concept [32]. Epistemological beliefs are considered as character traits and are domain-specific. Sophisticated epistemological beliefs, such as those related to the medical domain in particular, have been shown to influence learning strategies, learning outcomes [33], and information seeking behavior including source choice [32].

Finally, academic self-efficacy, meaning a belief in one’s own competence to be able to work effectively in an academic context [34] also has an impact on how people deal with scientific information and, thus, may also play a role in understanding the tentativeness of medical research findings. Academic self-efficacy is associated with personality-type variables such as social orientation or proactive personality, as well as with motivational factors [35,36] and learning-related emotions [37]. Given that academic self-efficacy specific to the science domain has been shown to correlate with actual academic success in science subjects [37,38], we assume an influence of this factor on the ability to grasp the tentativeness of medical research findings.

Previously, scientific literacy, epistemological beliefs, and academic self-efficacy have all been associated with the ability to apply critical thinking in traditional psychological experiments [22,39,40]. They have not been investigated concurrently, however, and even less with respect to influences on the critical evaluation of medical research findings on an online platform.

In addition to these three personality factors, we were also interested in the impact of a potentially relevant situational aspect because in Web-based discussion forums people are not at all limited to the mere critical reception of information. Interactive functions of Web 2.0 also allow active contributions, and so Web-based forums are typically characterized by a vigorous exchange of opinion, personal support, and guidance [41-43]. Users also frequently make reference to preceding posts in their contributions [16,44]. We therefore expect that when given the opportunity to post in a user forum dealing with medical research, people will be influenced by the comments that have already been posted by other forum users. It is well known that users tend to adjust their own contributions to that of others [45], and we assume that they will also adjust their own contributions according to the extent to which the issue of tentativeness has been addressed in preceding user comments. A previous analysis of traditional print media found that the perception of medical tentativeness depended on the salience of that issue in the text [46]. A potential extension of this effect to online forum settings is worth studying. Given the popularity of online platforms, a systematic investigation of the dynamic and multidirectional processes that result from effects of situational aspects of such salience of information is overdue.

### This Study

In a laboratory study, we presented students, who were laypeople with respect to the neurosurgical procedure of deep brain stimulation (DBS), with an online medical news article on that procedure. DBS involves the implantation of remote-controlled electrodes into the brain and has been used in experimental studies to alleviate symptoms of depression. Although its effectiveness is promising, it remains as yet unproven [47]. DBS was selected as the topic of the article because findings from studies of the procedure are characterized by great inconsistencies and an overall high level of tentativeness [47-49]. In addition, prior knowledge of DBS is uncommon among laypeople [46]. Also, based on a relatively high prevalence of depression in the population [50], any findings on the effectiveness of the procedure may be very relevant to a substantial proportion of the general public. Specifically, the article used in this study described a patient suffering from a severe case of depression, for whom DBS led to the restoration of a “normal” life. An online forum with commenting function associated with the article was simulated for the study.

We examined the relationships between scientific literacy, epistemological beliefs, academic self-efficacy, and people’s understanding of the tentativeness of the findings reported in the article, as well as potential effects of topics from preceding users’ comments on that same outcome. We examined participants’ perceived tentativeness of the case study findings reported in the online medical news article (measured by a questionnaire) and the extent to which they took tentativeness into consideration in their own comments in the forum (addressed tentativeness).

We posited the following hypotheses:

H1: Greater scientific literacy will enhance perceived tentativeness (H1a) and addressed tentativeness (H1b).

H2: More sophisticated epistemological beliefs in the medical domain will enhance perceived tentativeness (H2a) and addressed tentativeness (H2b).

H3: Greater academic self-efficacy in the science domain will enhance perceived tentativeness (H3a) and addressed tentativeness (H3b).

H4: Addressed tentativeness will depend on the degree to which comments by other users also address this issue.

Relationships between perceived tentativeness and addressed tentativeness were also explored.

## Methods

### Study Design

In a randomized controlled experiment, participants were randomly allocated to one of three experimental conditions. In all three conditions, they were asked to write a comment in response to an online newspaper-style text, elaborating on their views as extensively as possible. Conditions differed in terms of the “user” comments purportedly made previously, which participants could see in the simulated online forum. In control condition A, no comments were present. In condition B, previous comments were present but did not address the tentativeness of the findings reported in the article. In condition C, previous comments addressed the issue of tentativeness. Scientific literacy, epistemological beliefs, and academic self-efficacy were the trait variables measured. The outcome variables identified and measured were perceived tentativeness and actively addressed tentativeness in participants’ own comments.

### Sample

We recruited 70 participants aged 18-35 years from a university-wide pool of volunteers who confirmed upon study entry that they had no prior knowledge of DBS; 19 participants were male (27.1%) and 49 were female (70.0%). Two participants chose not to disclose their sex. Data were complete for all other measured variables. All participants were university students and all except one had obtained general qualification for university entrance. None of these participants was excluded from the study. Twenty-four participants were randomly assigned to condition A, 23 to condition B, and 23 participants to condition C. All gave full written informed consent and received 6€ for participating in a session that lasted approximately 45 minutes.

### Materials and Instruments

All participants received the same online newspaper-style article reporting a case study of a female patient with depression. The article was constructed in part on the basis of actual newspaper articles; the case study itself was fictional. It described a case in which a patient, following unsuccessful treatment with psychotropic drugs and psychotherapy, experienced substantial improvements in her quality of life after the application of DBS. The article was fabricated for the study in the style typical of mainstream media. It pointed out that the use of DBS in the patient was only experimental but did not explicitly stress the tentativeness of the content in the report.

Subsequent to reading the newspaper article, participants in the two comments conditions saw five fictitious comments by previous “users.” The “user” comments were designed to be similar in both groups in terms of word count (total of 276 and 306 words across comments for conditions B and C respectively). In addition, the comments were identical with regard to the (relatively neutral) attitude toward DBS that was expressed. The comments in both groups differed only in that for condition B the comments focused on the content of the text without addressing the tentativeness of the findings (eg, by relating the case study to the user’s own experiences with depression). For condition C, the five comments explicitly discussed the issue of tentativeness (eg, by pointing out that long-term effects of DBS were not identifiable on the basis of the case study, that the success described in the study may be due to chance or placebo effects, or that alternative explanations for the patient’s improved condition might exist).

We performed a test of the experimental material after completion of the main study to confirm that our manipulation of the level of tentativeness conveyed by the comments had been successful. For this purpose, 24 university students aged 19-30 years (mean age 22.4 years [SD 3.0]; 83% female) were presented with all 10 comments in random order and for each rated their agreement with the statement, “The comment communicates that the findings are preliminary and should be interpreted with care” on 7-point Likert scales. The five comments in condition B received an average rating of 1.53 (SD 0.39); those of condition C received an average rating of 5.35 (SD 0.63). As expected, the sum scores across the five comments for condition B (mean 7.67, SD 1.95) were significantly lower than for condition C (mean 26.75, SD 3.17), *t*_23_=24.32; *P*<.001. This shows that our manipulation was indeed successful as different levels of tentativeness were conveyed by these two groups of comments.

A total of four questionnaires were administered. Participants’ level of scientific literacy was measured by the Nature of Science Assessment (NoS), which has been used previously to measure scientific literacy in a student sample [51]. For each of the 7 items in the questionnaire, 4 response options were displayed. Single correct responses with no other options ticked were counted and contributed to a sum score (possible range 0-7).

Epistemological beliefs specific to the medical domain were assessed using the 24-item Connotative Aspects of Epistemological Beliefs (CAEB) Questionnaire [52]. On 7-point semantic differential scales, pairs of adjectives represented simpler versus more sophisticated beliefs in response to the statement, “Medical knowledge of psychiatric and psycho-motor diseases and their treatment is…” Scores were reversed as appropriate and summed, before the scale was adjusted to start at “0” (possible range of 0-144; higher scores indicating more sophisticated epistemological beliefs).

The level of participants’ academic self-efficacy in the science domain was measured using a 4-item scale [53], which in similar form has been shown previously to have good internal consistency [54]. Responses were made on 5-point Likert scales spanning from “do not agree at all” to “completely agree” (see Table 1). Scores were reversed as appropriate, before sum scores were calculated and the scale was adjusted to start at “0” (possible range 0-16).

**Table 1:** Items measuring academic self-efficacy.

Number	Item
1	“I am usually able to understand scientific content.”
2	“If I put enough effort into it, I succeed in gaining a good overview of the natural sciences.”
3	“If I have questions related to the field of science, I am usually able to help myself.”
4	“Without help, I am not able to deal with scientific topics at all.” (reversed item)

Participants’ perceived tentativeness of the findings presented in the journalistic article was measured by a 6-item scale (see Table 2), which has been used previously [46]. Agreement with each of the items was rated on 7-point Likert scales spanning from “not true at all” to “absolutely true.” Scores were reversed as appropriate and summed up. The scale was adjusted to start at 0, resulting in a possible score range of 0-36 (higher values indicating higher perceived tentativeness).

**Table 2:** Items measuring perceived tentativeness.

Number	Item
1	“The findings of the study are not very definite.”
2	“On the basis of this study, our understanding of DBS in depression is not complete yet.”
3	“The study is conclusive.” (reversed item)
4	“The findings are reliable.” (reversed item)
5	“The study offers a solid basis on which to decide on the future use or non-use of DBS in depression.” (reversed item)
6	“The findings of the study should be seen as preliminary only.”

Addressed tentativeness in participants’ comments was coded independently by 2 raters who were naïve to the research questions and blind to the experimental conditions. Prior to viewing the comments, a list of aspects of tentativeness relating to the newspaper article was set up and used to rate the extent to which tentativeness was addressed by the participants in their comments (score 0-6; resulting from one point for each of the 6 following aspects of tentativeness that were addressed by the participants: “uncertain long-term effects,” “single case study,” “lack of control condition,” “potential for placebo effect,” “inability to draw conclusions on all patients with depression on basis of study,” “need for further studies”). Before rating the participants’ comments, the 2 raters first became familiarized with the scoring procedure using five training comments. Following completion of the coding of all 70 comments for addressed tentativeness by the 2 raters and subsequent calculation of interrater reliability (see below), the average of the scores assigned by the raters was calculated for each comment and was used for the purpose of analyses.

Demographic information was obtained using a standard self-report questionnaire with items on age, sex, and the main subject of study.

### Procedure

The study was performed on laptops in the laboratory. Initially, participants read the newspaper article presented on the screen in their own time. On the next screen, the “user” comments on the article were presented for the two comments conditions (B and C) in a simulated forum (condition A did not receive any “user” comments), and participants in all three conditions were asked to write their own comments in a space provided on that same page. Instructions were non-specific, with the request to simply comment on the article in any way. No time limits or limits to a word count were imposed. Subsequently, the tentativeness questionnaire, the NoS scale, the CAEB scale, and the academic self-efficacy questionnaire, as well as demographic information were completed.

### Statistical Analysis

Scales were initially assessed for internal consistency, and interrater reliability for addressed tentativeness scores was calculated. Bivariate Pearson correlations explored associations among all predictor and outcome variables. In order to investigate the relationship of the trait variables with perceived and addressed tentativeness scores, a linear regression model was calculated for each of the two outcome measures. Scientific literacy (NoS), epistemological beliefs (CAEB), and academic self-efficacy scores were all entered concurrently into the models in order to evaluate the independence of associations. Each of the two models controlled for experimental condition. The model on addressed tentativeness additionally controlled for word count on the basis that participants who wrote longer comments had a relatively greater chance of obtaining high scores on that measure compared with participants who wrote shorter comments. Finally, an analysis of covariance (ANCOVA) with adjustment for word count and for all three trait variables compared addressed tentativeness scores among the three experimental conditions. All statistical analyses were two-tailed.

### Ethical Approval

The study had ethical approval from the Institutional Ethics Committee (approval reference: LEK 2014/001).

## Results

### Scales and Sample Characteristics

Internal consistencies for the CAEB (Cronbach alpha=.88), academic self-efficacy (alpha=.86), and perceived tentativeness (alpha=.74) scales were found to be good. Agreement between the 2 raters on addressed tentativeness was very good (intraclass correlation coefficient=.90). The average of the addressed tentativeness score was negatively skewed, with 24 participants (34.4%) receiving a score of 0, meaning that these individuals did not address the issue of tentativeness in their comments at all. Nonetheless, we decided not to transform the variable, given that the transformation of count data such as these may lead to a bias in results when used in parametric analyses [55]. All of the remaining variables were normally distributed. Overall sample characteristics are presented in Table 3.

**Table 3:** Sample characteristics.

Characteristics		Values and scores
Age (years), mean (SD)		24.50 (3.95)
Female sex, n (%)		49 (70.0)
**Subject of study, n (%)**		
	The humanities	23 (32.9)
	Natural sciences	10 (14.3)
	Pedagogics	6 (8.6)
	Economics	6 (8.6)
	Law	6 (8.6)
	Other	19 (27.1)
Scientific literacy (NoS) (possible range 0-7), mean (SD)		2.36 (1.46)
Epistemological beliefs (CAEB) (possible range 0-144), mean (SD)		75.41 (16.38)
Academic self-efficacy (possible range 0-16), mean (SD)		11.09 (2.88)
Perceived tentativeness (possible range 0-36)		24.07 (5.57)
Addressed tentativeness (average of 2 raters; possible range 0-6), median (IQR)		1.0 (0.0-2.0)
Comment word count (range 38-383), mean (SD)		165.0 (73.2)

### Evaluation Outcomes

Two-tailed Pearson correlation analyses (see Table 4) revealed that participants who obtained higher scientific literacy scores referred to tentativeness to a greater extent in their comments. A higher epistemological beliefs score was associated with higher perceived tentativeness, and higher academic self-efficacy was linked to greater addressed tentativeness. The positive association of addressed tentativeness with perceived tentativeness was relatively modest, though highly significant.

**Table 4:** Pearson correlations among measured variables.

	*r* (*P* value)					
	Age	Scientific literacy	Epistemological beliefs	Academic self-efficacy	Addressed tentativeness	Perceived tentativeness
Scientific literacy	.09 (.474)					
Epistemological beliefs	.10 (.433)	.07 (.583)				
Academic self-efficacy	.03 (.794)	.12 (.335)	-.09 (.480)			
Addressed tentativeness	.18 (.134)	.24 (.017)^a^	-.03 (.798)	.28 (.019)^a^		
Perceived tentativeness	.25 (.035)^a^	-.03 (.828)	.25 (.037)^a^	.14 (.406)	.39 (.001)^a^	
Word count	.05 (.698)	-.05 (.668)	-.01 (.932)	.01 (.962)	.22 (.063)	.10 (.416)

^a^ *P* values are significant.

In order to further investigate the findings from the univariate analyses presented in Table 4, we calculated two linear regression models. For each of the two outcomes (perceived tentativeness and addressed tentativeness), all predictors were entered concurrently into a single model that controlled for experimental condition and—in the case of addressed tentativeness—also for word count (see Table 5). Epistemological beliefs were identified as a statistically significant predictor of perceived tentativeness, with higher CAEB scores associated with greater perceived tentativeness. This association supported H2a and was independent of participants’ scientific literacy, their academic self-efficacy, and experimental condition. The reverse pattern of findings was observed for addressed tentativeness. Here, both higher scientific literacy (H1b) and higher academic self-efficacy (H3b) predicted a more elaborately addressed tentativeness in participants’ comments; these effects were independent of one another and of experimental condition and word count. The remaining hypothesized associations (H1a, H2b, H3a) were not supported by these analyses.

**Table 5:** Models of perceived tentativeness and addressed tentativeness on scientific literacy, epistemological beliefs, and academic self-efficacy^a^.

	Perceived tentativeness		Addressed tentativeness	
	Standardized *β* (standard error)	*P* value	Standardized *β* (standard error)	*P* value
Scientific literacy	-.06 (.46)	.603	.25 (.09)^b^	.025
Epistemological beliefs	.26 (.04)^b^	.034	-.05 (.01)	.631
Academic self-efficacy	.14 (.24)	.238	.31 (.05)^b^	.007

^a^Findings from two linear regression models (for perceived and addressed tentativeness, respectively) with all predictor variables entered in a single step. Both models controlled for experimental condition; the analysis of addressed tentativeness additionally controlled for word count. Total *r* ^*2*^for model of perceived tentativeness=.08. Total *r* ^*2*^for model of addressed tentativeness=.27.

^b^Values are significant.

In order to evaluate the effect of experimental condition on addressed tentativeness, an ANCOVA with adjustment for the word count of the participants’ own comments as well as for their scientific literacy, epistemological beliefs, and academic self-efficacy assessed mean addressed tentativeness in the three experimental conditions. As expected in H4, there was an overall effect of condition on addressed tentativeness, *F*_2,63_=3.66; *P*=.049, η_p_^2^=.092. Post-hoc pairwise comparisons revealed that this effect was driven by a statistically significant difference in addressed tentativeness between the control condition A and condition C where the preceding comments discussed tentativeness; adjusted means in condition A 0.73, 95% CI 0.28-1.18; standard error 0.23 versus adjusted means in condition C 1.54, 95% CI 1.09-1.99; standard error 0.23; *P*=.015. The remaining pairwise comparisons of group differences in addressed tentativeness did not reach statistical significance (both *P*>.10).

## Discussion

### Main Findings

This study investigated the roles that science-related interindividual character differences and themes discussed in other people’s online forum contributions play in the critical evaluation of an online medical news article by students who were laypeople with respect to the topic of the article. To our knowledge, this is the first study to assess the extent to which users discuss medical tentativeness in their comments on a medical topic in a simulated online forum.

Results of the measurements carried out in the study showed that participants with greater scientific literacy and those with higher academic self-efficacy actively addressed the issue of tentativeness to a relatively greater extent than participants who had lower scores in these dimensions. Importantly, the findings for scientific literacy and academic self-efficacy were independent of one another. Epistemological beliefs were not a predictor of addressed tentativeness. However, in line with our expectation, we found evidence showing that participants who believed medical knowledge to be relatively more complex (ie, had more sophisticated epistemological beliefs) perceived the tentativeness in the article’s research findings to a higher degree. Neither scientific literacy nor academic self-efficacy was related to perceived tentativeness (it should be noted here that in a previous study [56] general self-efficacy—not academic self-efficacy—was even negatively associated with perceived tentativeness).

The finding that people with more sophisticated epistemological beliefs demonstrate a greater ability to detect tentativeness in medical research is consistent with the literature on epistemological beliefs and critical thinking in science in general [39] and in the domain of medical research [46]. On the basis of this association in the literature in the medical research domain in particular, our expectations extended to an effect of epistemological beliefs on the degree to which the tentativeness issue was actively addressed in users’ own comments. This was found not to be the case in the sample we used for this study, and we can only speculate as to potential underlying reasons. It is possible that people with more sophisticated epistemological beliefs did successfully identify tentativeness in the less demanding information processing that occurred in answering questions in a questionnaire but did not make any effort to actively engage in the discussion of the issue in their own contributions.

The association of higher scientific literacy and higher academic self-efficacy with greater addressed tentativeness is consistent with previous investigations that had identified relationships of these factors with critical thinking ability [28,40]. We have extended this previous evidence by showing that this same association applies to the active evaluation of medical research in an online forum. Higher academic self-efficacy and greater scientific literacy may have each promoted deeper levels of processing of the information that was provided in the article. The absence of a correlation between scientific literacy and academic self-efficacy was somewhat surprising, considering that both essentially measured scientific knowledge. A degree of disparity between the self-perception of ability (academic self-efficacy) and actually measured ability (scientific literacy) has frequently been reported in the research literature on self-perception [57] and may be a plausible explanation for the lack of this correlation in our study.

We had also expected that participants who were exposed to comments already appearing in the forum that addressed the issue of tentativeness would be influenced in the content of their contributions. A difference was indeed found between the experimental conditions insofar as participants in the condition receiving comments that mentioned tentativeness (condition C) scored higher on addressed tentativeness compared to the participants in the control condition (condition A). With this finding, we have extended previous evidence that had identified salience of tentativeness in a journalistic article as predictive of perceived tentativeness [46], by demonstrating a similar effect of that salience when it is presented in user comments in an online forum. The relatively modest size of the effect may have been due to the fact that complex processes were at play. For instance, reading existing comments by other users that addressed the issue of tentativeness may have led to individuals’ wishing to discuss that same issue too, but at the same time may have decreased their discussion due to the notion that tentativeness had already been addressed sufficiently by other users.

### Limitations, Future Work, and Recommendations

In the past, online forum posts on medical topics have mainly been used for thematic language analyses [19]. There is little control over potential influential factors and ethical issues associated with the lack of informed consent in actual online forums [58]. Our use of a simulated forum for comment has now avoided these ethical issues by enabling us to obtain consent by participants [59]. Moreover, it allowed the experimental manipulation of a specific situational aspect as well as the determination of what impact personality variables would have that are usually obscured in the anonymity of the World Wide Web. Our findings are further strengthened by the use of a topic that participants had no prior knowledge or opinion of, ruling out any effects by interindividual differences in these aspects on their activity in the simulated forum.

However, the somewhat artificial setting of the forum that included researcher-generated comments and text-based experimental manipulation in a single session is a potential shortcoming of this study. Our findings are further limited by the fact that analyses were based on a sample of university students. Future laboratory studies should consider using non-specific samples in order to determine whether the effects identified here extend to the general, including non-student, population. Around one third of the student sample presented here failed to address tentativeness at all in their comments, despite being equipped with at least basic scientific education in secondary school that should provide them with the capacity to deal with scientific material and with the aptitude to apply critical thinking. Accordingly, laypeople in the general population would be expected to address tentativeness even less than was reported here.

We further used only one topic in the current study (DBS), and our findings may not generalize to other scientific topics, including medical research topics. It may be the case that the effects seen in this study in fact would be different if participants were to deal with tentative research findings in other domains. Future studies should therefore aim to extend the present results by evaluating other scientific topics. Nonetheless, by focusing on DBS, we have highlighted a field of medical research that may warrant particular care in the communication of research findings to the general population. Researchers working on DBS and who interact with science journalists and university media outlets may be advised to consider the online dissemination of their findings. Online forums in particular may represent one useful way to influence the perception of DBS research and to increase knowledge in the general population of this often life-saving procedure.

In addition to the manipulation of other possible situational factors that could influence people’s behavior in online forums, future studies should make use of real-life forums to investigate health-related thinking and behavior in patients and laypeople [60]. Findings from such studies could then directly feed into applications such as postgraduate courses, which could create a bridge between science and the media [3].

Finally, future research should also take a variety of control variables into account that might have an impact on how laypeople deal with tentative scientific information. Such control variables might include people’s personal interest in medical issues in general or in the particular medical topic at hand; whether they have been diagnosed with a relevant disease (here, depression), or in the case of more established therapeutic treatments, their prior knowledge of the medical procedure.

### Conclusions

The processes involved in laypeople’s active contribution to online medical forums are highly complex and dynamic and are therefore difficult to investigate systematically. The study reported here has made an advance by applying a simulated online forum in a controlled laboratory setting. This allowed for the manipulation of situational aspects as well as the precise measurement of trait factors that may influence laypeople’s behavior in such a forum. We have shown that the ability to understand the tentativeness of DBS research in an online forum was not at all universal. Influential factors included people’s scientific literacy, epistemological beliefs, and academic self-efficacy. Their understanding of tentativeness was additionally and independently also affected by other users’ comments already appearing in the simulated forum.

We have made an observation that calls for awareness in future investigations, particularly in those involving real-life online forums. Users appear to be only as “good” at applying critical thinking as the existing system of the forum itself. In order to recognize the tentativeness of medical research findings, readers of online medical news articles do not only depend on article authors to refer to the fact that research findings may be uncertain, temporary, controversial, or inconsistent [61]. They may also benefit from other Internet users who have already gained this insight and have identified the scientific tentativeness in their own comments.

Targeting the specific trait characteristics that were identified in this study as being influential on the ability to understand the tentativeness of medical research, and which are modifiable to greater or lesser degrees [34,62,63], may be a fruitful approach. Specifically, scientific literacy, epistemological beliefs, and academic self-efficacy could all represent useful targets for modification through formal instruction. Promoting scientific literacy and supporting people in recognizing the nature of scientific knowledge may support the public understanding of science. Such educational programs should include addressing how people deal with scientific topics, how they understand knowledge itself, and how they should deal with individuals’ motivations for advancing particular views [64,65]. Programs for intervention that target these factors may well have the potential to promote critical thinking in laypeople who participate in online forums to discuss findings on DBS and potentially other medical research findings [66].

## Abbreviations

ANCOVA: analysis of covariance
CAEB: Connotative Aspects of Epistemological Beliefs
DBS: deep brain stimulation
NoS: Nature of Science
